# Supplementary material for: Glaucoma Detection Using Support Vector Machine Method Based on Spectralis OCT
Source: Diagnostics (Basel). 2022 Feb 3;12(2):391. doi: 10.3390/diagnostics12020391 (PMC8871188; doi:10.3390/diagnostics12020391)
Supplement: Supplementary file 1 [file diagnostics-12-00391-s001.zip › diagnostics-1528397-supplementary.pdf]

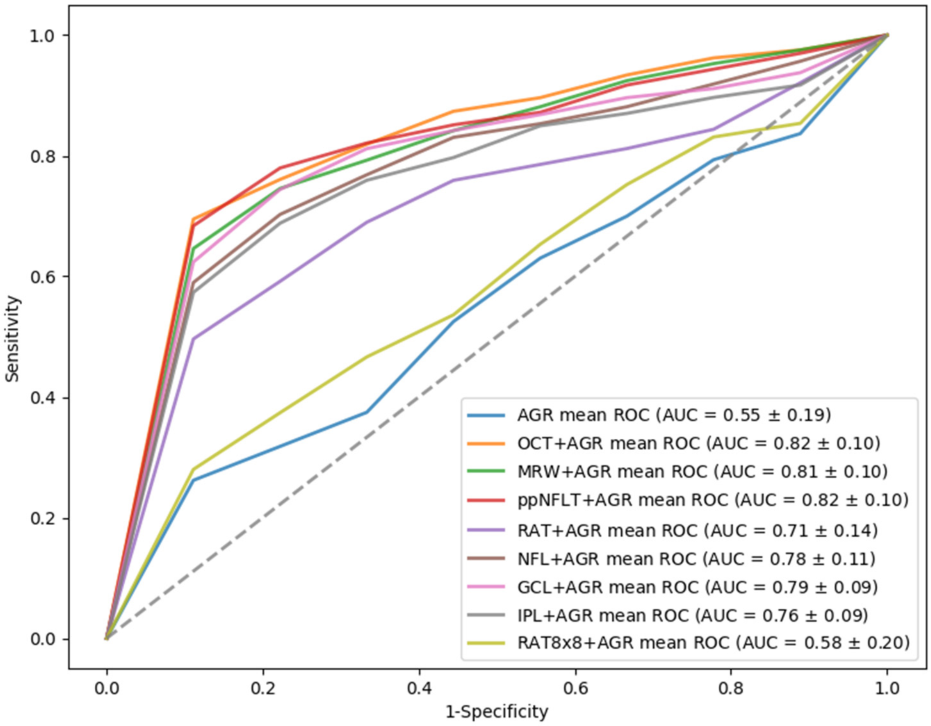

Figure S1. Receiver operating characteristic (ROC) curve of optical coherence tomography (OCT)-related features groups plus age, gender and refraction in differentiating normal from glaucomatous eyes

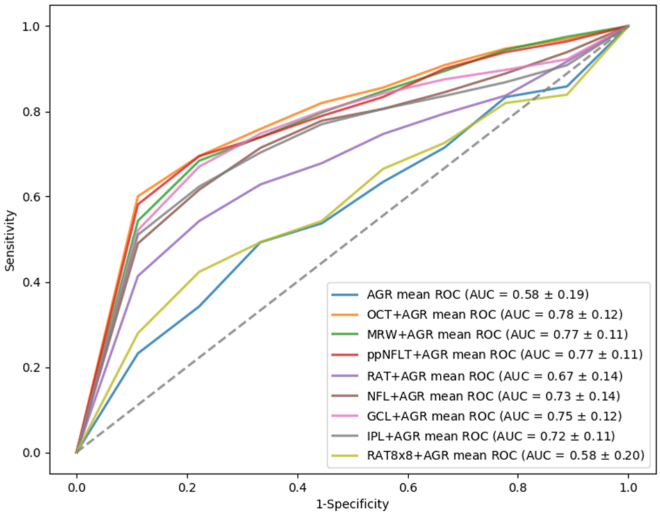

(a)

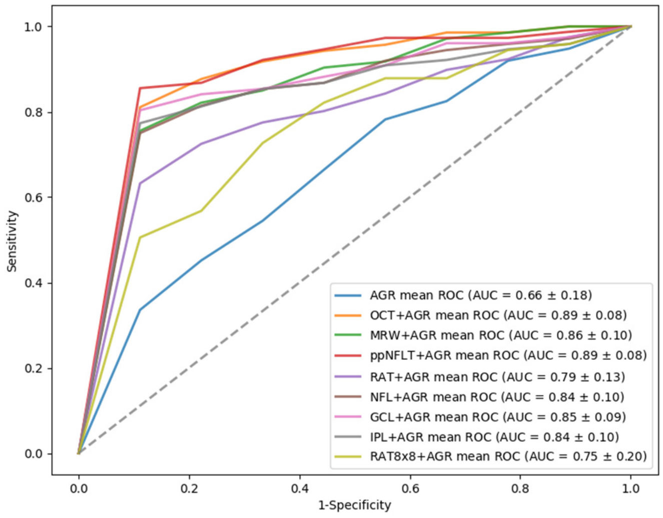

(b)

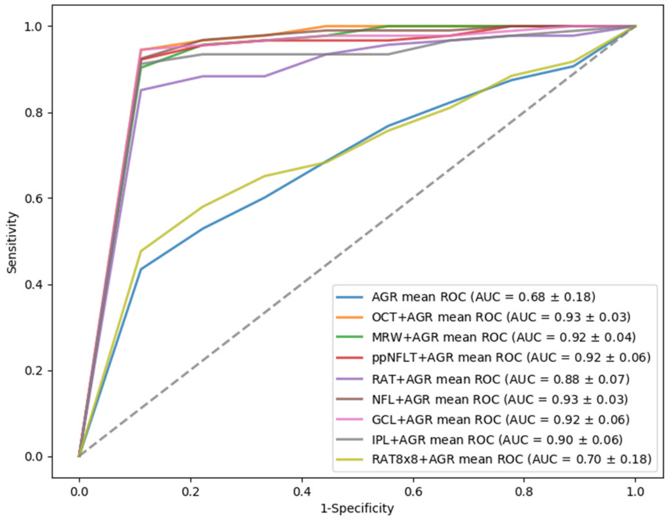

(c)

Figure S2. Receiver of characteristic (ROC) curve of optical coherence tomography (OCT)-related features groups plus age, gender and refraction in differentiating normal from **(a)** early, **(b)** moderate, and **(c)** severe glaucomatous eyes
